# Tuberculous meningitis: new tools and new approaches required

**DOI:** 10.12688/wellcomeopenres.15591.1

**Published:** 2019-11-19

**Authors:** James A Seddon, Guy E Thwaites, Tuberculous Meningitis International Research Consortium, Rob E. Aarnoutse, Rob E. Aarnoutse, Suzanne T. B. Anderson, Suzanne T. B. Anderson, Nathan C. Bahr, Nguyen D. Bang, David R. Boulware, Tom Boyles, Lindsey H. M. te Brake, Satish Chandra, Felicia C. Chow, Fiona V. Cresswell, Reinout van Crevel, Angharad G. Davis, Sofiati Dian, Joseph Donovan, Kelly E. Dooley, Anthony Figaji, A. Rizal Ganiem, Ravindra Kumar Garg, Diana M. Gibb, Raph L. Hamers, Nguyen T. T. Hiep, Darma Imran, Akhmad Imron, Sanjay K. Jain, Sunil K. Jain, Byramee Jeejeebhoy, Jayantee Kalita, Rashmi Kumar, Vinod Kumar, Arjan van Laarhoven, Rachel P-J. Lai, Abi Manesh, Suzaan Marais, Vidya Mave, Graeme Meintjes, David B. Meya, Usha K. Misra, Manish Modi, Alvaro A. Ordonez, Nguyen H. Phu, Sunil Pradhan, Kameshwar Prasad, Alize M. Proust, Lalita Ramakrishnan, Ursula Rohlwink, Rovina Ruslami, Johannes F. Schoeman, James A. Seddon, Kusum Sharma, Omar Siddiqi, Regan S. Solomons, Nguyen T. T. Thuong, Guy E. Thwaites, Ronald van Toorn, Elizabeth W. Tucker, Sean A. Wasserman, Robert J. Wilkinson

**Affiliations:** 1 Infectious Diseases, Imperial College London, London, W21PG, UK; 2 Desmond Tutu TB Centre, Department of Paediatrics and Child Health, Stellenbosch University, Cape Town, Western Cape, 7505, South Africa; 3 Centre for Tropical Medicine and Global Health, Nuffield Department of Medicine, University of Oxford, Oxford, UK; 4 Oxford University Clinical Research Unit, University of Oxford, Ho Chi Mihn City, Vietnam

**Keywords:** Tuberculous meningitis, TBM, Tuberculosis, Consortium

## Abstract

Tuberculous meningitis is the most severe form of tuberculosis and causes widespread mortality and morbidity. Understanding of the epidemiology and pathogenesis is incomplete, and the optimal diagnosis and treatment are poorly defined. To generate research collaboration and coordination, as well as to promote sharing of ideas and advocacy efforts, the International Tuberculous Meningitis Research Consortium was formed in 2009. During the most recent meeting of this group in Lucknow, India, in March 2019, the Consortium decided to bring together key articles on tuberculous meningitis in one supplement. The supplement covers recent scientific updates, expert perspectives on specific clinical challenges, consensus statements on how to conduct research, and a set of priorities for future investigation.

## Editorial

Tuberculous meningitis (TBM) is the most severe form of tuberculosis (TB) and it predominantly affects young children and individuals living with human immunodeficiency virus (HIV). If left untreated it is universally fatal, but even with prompt diagnosis and treatment around one fifth die, and over half of survivors have significant neurological deficit ^1^. In adults with advanced HIV, mortality from TBM exceeds 50% ^2^; and when the disease is caused by bacteria resistant to rifampicin and isoniazid death results in almost all ^3– 5^.

The global burden of TBM is unknown. Many individuals with TBM are never diagnosed, treated or reported to surveillance systems. In cohorts of individuals treated for TB, the proportion with TBM varies, dependent on context, TB and HIV prevalence, demographic characteristics of the cohort, diagnostic capabilities and location. It is likely that 2–5% of TB cases are TBM and given a global burden of over 10 million incident TB cases each year ^6^, as many as half a million individuals may develop TBM worldwide each year.

TBM presents some unique challenges to those trying to understand it better and improve outcomes. First, the disease is hard to diagnose, primarily because the symptoms and signs are non-specific and the numbers of bacteria in the cerebrospinal fluid (CSF), the essential diagnostic specimen, are very low and difficult to detect. Second, the anti-TB drugs used to treat TBM vary in their ability to cross the blood-CSF and blood-brain barriers and kill the bacteria. Third, the intra-cerebral inflammatory response may help control the replication and dissemination of bacteria within the brain, but also leads to brain tissue ischaemia and infarction, raised intracranial pressure, and mass effects caused by space occupying lesions. Fourth, whilst the killing of bacteria and the control of inflammation are achieved, the critical care of a patient with TBM needs attention, especially the detection and treatment of raised intracranial pressure. Taken together, TBM presents a special set of challenges that require a coordinated and concerted effort, through research, clinical practice, and public health.

TBM has been a neglected field of TB research with many substantial knowledge gaps. Understanding of the epidemiology is limited, with poor appreciation of the global disease burden, reducing advocacy and resource allocation. The currently available laboratory diagnostic tools have poor sensitivity, leading to many missed cases, while clinical diagnostic approaches lack both sensitivity and specificity, resulting in over- and under-treatment. We are a long way from a complete understanding of the best drug therapies to kill mycobacteria and the best approaches to modify the host immune response. Understanding of TBM pathogenesis is sub-optimal, leading to challenges in identifying molecular pathways and host responses that might be targeted by new drugs. The optimal critical care required to support patients with TBM has not been systematically investigated and the best health system strategies to minimise loss during the TBM care cascade are poorly defined.

Globally there are relatively few individuals carrying out studies in TBM and few centres of excellence in TBM research exist. The field would therefore benefit from co-ordination, advocacy, collaboration and early data sharing. To this end, the TBM International Research Consortium was first convened in Cape Town, South Africa, in 2009. A group of 50 researchers met to share experiences and early research findings; a key output from that meeting was the development of a uniform TBM case definition for use in clinical research ^7^. A second meeting was organised in Dalat, Vietnam in 2015, from which came standardised methods for the conduct of TBM clinical studies ^8^, a perspective on the use of GeneXpert in TBM diagnosis ^9^, and a state-of-the-art review ^10^. At the most recent meeting on 1–2 March 2019, in Lucknow, India, the consortium decided to write a series of articles, published in one supplement, covering the most up-to-date developments in every aspect of TBM research, including pathogenesis, diagnostics, drug development, pharmacokinetics and host directed therapy. Expert reviews on optimal clinical care in certain specific and challenging circumstances were also included, namely the management of hyponatraemia and tuberculomas. The consortium decided to develop a checklist to guide the supportive and critical care of individuals with TBM and produce consensus statements on the conduct of TBM research to encourage consistency and comparability of data. This included articles on how to take samples in research studies and how to undertake neurocognitive assessments. A review of the care cascade in TBM within health systems had not been previously been conducted, a necessary first step in identifying gaps, therefore an article on this topic was suggested. Finally, the consortium concluded that documenting knowledge gaps and research priorities would help to focus future work for researchers and funders. A key requirement for the supplement was that it was open access and widely available, leading to the decision to submit to Wellcome Open Research. 

Far too many people continue to suffer the consequences of TBM each year and the global community has been slow to respond. We hope that the articles published within the supplement will describe what is currently known about TBM, identify the major knowledge gaps and research priorities, and promote and inspire the necessary collaborative research required to address them and thereby reduce the numbers of lives lost or impaired by this devastating disease.

## Data availability

### Underlying data

No data are associated with this article
